# An effective white spot syndrome virus challenge test for cultured shrimp using different biomass of the infected papilla

**DOI:** 10.1016/j.mex.2019.07.007

**Published:** 2019-07-10

**Authors:** Cristóbal Domínguez-Borbor, Irma Betancourt, Fanny Panchana, Stanislaus Sonnenholzner, Bonny Bayot

**Affiliations:** a Escuela Superior Politécnica del Litoral, ESPOL, Centro Nacional de Acuicultura e Investigaciones Marinas, CENAIM, Campus Gustavo Galindo Km 30.5 Vía Perimetral, P.O. Box 09-01-5863, Guayaquil, Ecuador; b Escuela Superior Politécnica del Litoral, ESPOL, Facultad de Ingeniería Marítima y Ciencias del Mar, FIMCM, Campus Gustavo Galindo Km 30.5 Vía Perimetral, P.O. Box 09-01-5863, Guayaquil, Ecuador

**Keywords:** WSSV challenge test using different biomass of infected-muscle, *Per os* challenge test, Dose-response, Virus virulence, WSSV

## Abstract

White spot syndrome virus (WSSV) is one of the most virulent pathogens of cultured penaeid shrimp. Several control strategies are used commonly to mitigate the economic losses caused by the pathogen, such as application of antiviral products at farm level. One of the most practical method for the screening of potential anti-WSSV products is through challenge tests. Therefore, it is essential to develop simple, reproducible and effective bioassays able to simulate specific mortality levels. The purpose of this study was to develop a simple and reproducible bioassay that simulate different mortality levels by varying the proportion of WSSV-infected and noninfected shrimp tissues administered to susceptible shrimp during a *per os* challenge test. This method mimics one of the natural transmission routes of WSSV infection in shrimp and could be applied to identify potential antiviral products to different cultured shrimp species susceptible to WSSV.

Here we report:

• A simple and economic method to evaluate therapeutic antiviral products against WSSV through a challenge test, that uses different biomass amounts of WSSV-infected papilla.
• Allows to simulate a wide and reproducible range of mortalities observed in shrimp farms.
• A challenge test that simulates one mode of natural WSSV infection in shrimp.

## Graphical abstract

**Figure:**
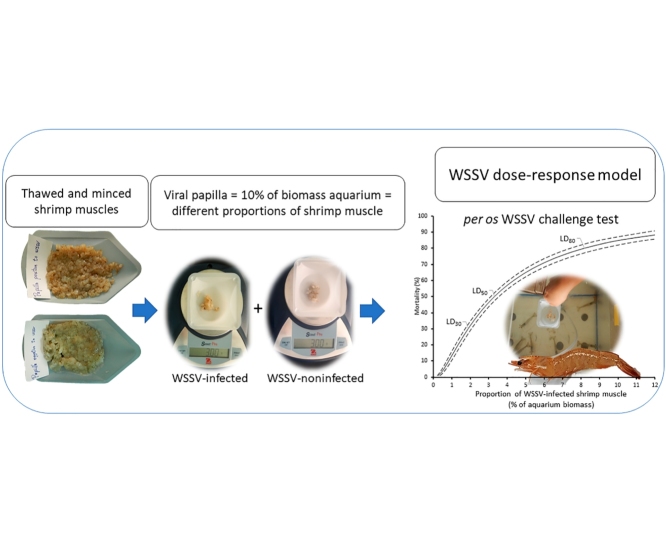

**Specification Table**

**Table:** 

Subject Area:	Veterinary Science and Veterinary Medicine
More specific subject area:	Shrimp health
Method name:	WSSV challenge test using different biomass of infected-muscle
Name and reference of original method:	Q. Wang, B.L. White, R.M. Redman, D. V. Lightner, *Per os* challenge of *Litopenaeus vannamei* postlarvae and *Farfantepenaeus duorarum* juveniles with six geographic isolates of white spot syndrome virus, Aquaculture. 170 (1999) 179–194. doi:10.1016/S0044-8486(98)00425-6.
Resource availability:	All information is available in the manuscript

## Overview

White spot syndrome virus (WSSV) is one of the most virulent pathogens reported for cultured penaeid species [1,2] still causing high mortalities and economical losses [[3], [4], [5]]. Challenge tests are used to evaluate potential control methods for shrimp diseases [[6], [7], [8], [9], [10]], of which, the *per os* challenge offering infected papilla to susceptible shrimp reproduces one of the pathways of shrimp WSSV infection in farms. However, these challenge tests have proven to exert variable mortalities that could be attributed to the non-uniformity of viral loads in the tissues offered during the tests [11,12]. Here, we report a reproducible and precise bioassay for the evaluation of antiviral products against WSSV through the standardization of the mortality responses to different amounts of WSSV-infected and noninfected shrimp tissues.

## Method details

### Materials

Biological material: WSSV-infected and non-infected homogenized shrimp tissues

Scalpel

Cutting board

Plastic weighing dishes

Aluminum foil

Paper tape

Permanent marker

Notebook

Microsoft Excel to display and analyze data

Statistical software to calculate lethal doses (LD)

### Reagents

Sodium hypochlorite (200 mg L^−1^)

### Equipment

Freezer (at −80 °C)

Digital scale, precision 0.01 g

Bioassay room with experimental units

### Experimental shrimp and rearing conditions

Healthy juveniles of *Penaeus vannamei* shrimp (3–8 g) are transferred to the test room and randomly allocated to aquariums at a density of 10 shrimp per 50-L aquariums. Each aquarium is filled with filtered and UV sterilized seawater, and continuously aerated. Food, molts and fecal material is daily siphoned out. Water exchange in tanks is set to 50% per day. Shrimp are fed daily with a commercial diet at 5% of their estimated biomass during a quarantine period of seven days to verify the absence of WSSV infection. Shrimp are starved for 24 h prior to the start of the challenge test.

### Viral papilla

Before the initiation of the challenge test, WSSV-infected and noninfected shrimp tissues (muscle) stored at −80 °C are thawed and minced with scalpels into small pieces (0.5–1.0 mm^2^). The viral papilla is prepared combining different proportions of both tissues to obtain four different viral papilla concentrations (Table 1). The amount of infected and non-infected tissue will total 10% of the shrimp biomass obtained in each aquarium (Table 1).

**Table 1:** Viral papillae prepared with WSSV-infected and noninfected shrimp tissues administered at 10% of the aquarium shrimp biomass.

Aquarium biomass proportion (%) of administered shrimp tissues	
WSSV-infected	WSSV-noninfected
1.5	8.5
2.5	7.5
5.0	5.0
10.0	0.0

### WSSV challenge

Shrimp are *per os* infected supplying (only one dose) the four viral papillae (treatments) in each aquarium (six replicates per treatment). For this, both tissues infected and non-infected at the correspondent proportions are homogenized and distributed to the aquariums. Shrimp pertaining to a double negative control group (six replicates) are fed with 10% shrimp biomass of WSSV-noninfected shrimp muscle. Shrimp of treatments and double negative control group are not fed with the commercial diets for the next 24 h and afterward, shrimp are fed with a commercial diet for nine days at 5% of its daily biomass.

### Mortality observation and confirmatory analysis

After 24 -hs post-exposure, mortality is recorded every 2 h during the peak of the mortality period, which is expected to occur within 96 h post-exposure, and every 4 h during the last days of the challenge test. Moribund shrimp are removed during the challenge to avoid re-infection. The challenge ends after 10 days post-exposure. A suggested sample of 10% of moribund shrimp from the infected treatments and survivors from the negative controls are collected for nested polymerase chain reaction (PCR) and histopathologic analysis, to confirm the existence or absence of infections and lesions in infected and noninfected treatments.

### WSSV dose-response model

**Fig. 1:**
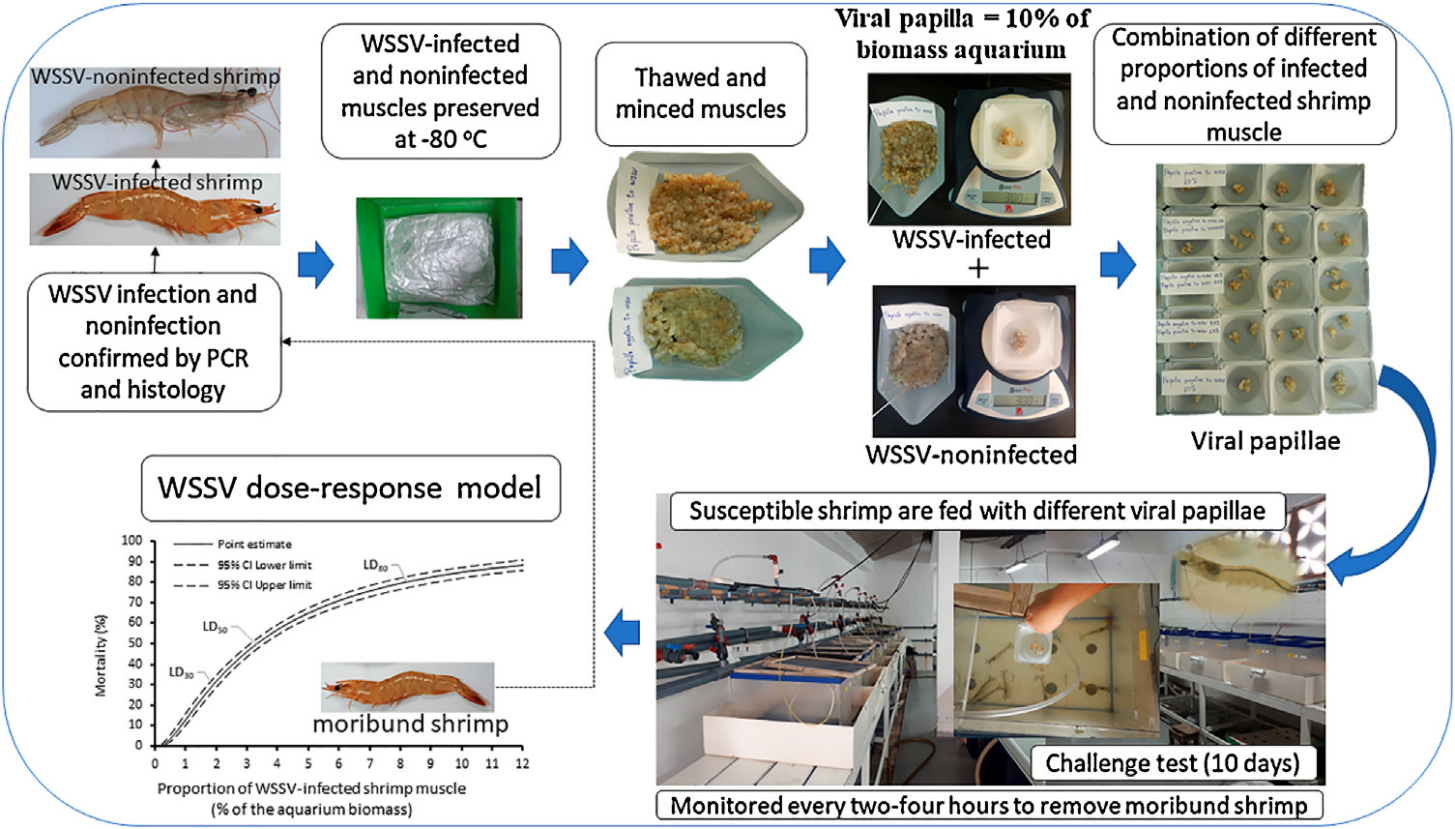
Pictorial representation for the process of WSSV challenge by offering different proportion of WSSV-infected papilla and WSSV dose-response model.

The WSSV dose–response relationship is described as the variability of shrimp cumulative mortality after 10 days post-exposure as a function of the proportion (% of aquarium biomass) of WSSV-infected shrimp muscle supplied during the challenge test (Fig. 1). Mortality data are modeled with a probit regression and lethal doses (LD) of the proportion of WSSV-infected shrimp muscle causing different mortality responses are obtained (Fig. 1).

### Validation method

Six challenge tests were carried out for the validation of the proposed WSSV dose-response model, with healthy shrimp weighing 3.0 ± 1.4 g (Challenge tests 1, 2 and 3) and 8.0 ± 1.2 g (Challenge tests 4, 5 and 6). Water temperature of tests ranged between 25 and 28 °C and concentration of dissolved oxygen was maintained above 5.0 mg L^−1^. Salinity for all challenge tests was 35.0 ± 0.0 g/L. Healthy juveniles of *P. vannamei* shrimp were obtained from a local shrimp farm (Santa Elena, Ecuador) and transferred to an experimental room at the Centro Nacional de Acuicultura e Investigaciones Marinas (CENAIM, Santa Elena, Ecuador). Shrimp were randomly allocated (10 shrimp per aquarium) to 50 L glass aquariums (experimental units). Each aquarium was filled with 40 L of filtered (1 μm) and UV sterilized seawater, and continuously aerated. Protocols of water exchange, feeding, viral papilla preparation, treatments, the double negative control group, and challenge tests were performed as previously described (Method details). All experimental units were managed in the same way.

The healthy status of the experimental shrimp (20 in average for each challenge) was confirmed by PCR and histopathologic analysis. PCR analysis were performed following the methodology described by Pradeep et al. [13]. For the histopathologic analysis, shrimp were fixed with Davidson AFA solution and tissues were processed according to the procedures outlined by Bell and Lightner [14]. The WSSV infection of the viral papillae was also confirmed by nested PCR analysis. PCR-real time analysis was used to quantify the WSSV load for the four viral papillae. For each challenge, 10% of the moribund shrimp were processed for WSSV confirmatory analysis by nested PCR and histopathologic analysis.

Differences in cumulative mortality at 10 days post-exposure among treatments were analyzed by one-way analysis of variance (ANOVA). The null hypothesis (no effect in proportions of WSSV-infected shrimp muscle used in the challenge tests) was rejected with a P-value of the F-test ≤ 0.05. Tukey’s Honest Significant Difference test was used to compare treatment means. Previously, variance homogeneity of all treatments was examined through the Bartlett test and assumption of normality were examined through the Shapiro-Wilk normality test. Dose-response data (mortality-proportion of WSSV-infected shrimp muscle) were modeled with probit regressions and lethal doses causing 30, 50 and 80% of mortality were estimated (LD_30_, LD_50_ and LD_80_) for shrimp of 3.0 and 8.0 g. Analysis of probit regression were performed using IBM SPSS Statistics Version 20. All six challenge tests were carried out following the procedure described above.

Significant differences among all proportions of WSSV-infected shrimp muscle were found in all six challenge tests (Table 2). At each proportion of WSSV-infected shrimp muscle supplied in the challenge tests, similar levels of mortalities within and between shrimp weight groups were found, with a maximum mortality difference of 6.7%, indicating that specific proportions induce similar and reproducible levels of mortality through time (Table 2). No mortality was observed in the double negative control group in any of the six challenge tests.

**Table 2:** Cumulative mortality (average ± standard deviation) of *P. vannamei* shrimp after 10 days of post-exposure in six challenge tests used for the calibration of the WSSV dose-response model.

Proportion of WSSV-infected shrimp muscle (% of aquarium biomass) used in the viral papillae	Shrimp weight (3.0 ± 1.4 g)		Shrimp weight (8.0 ± 1.2 g)	
	Number of challenge test (mean shrimp weight)	Cumulative mortality (%)	Number of challenge test (mean shrimp weight)	Cumulative mortality (%)
1.5	1 (2.8 ± 0.7 g)	16.7 ± 5.2 ^a^	4 (7.9 ± 0.5 g)	21.7 ± 7.5 ^a^
2.5		48.3 ± 7.5 ^b^		43.3 ± 5.2 ^b^
5.0		60.0 ± 6.3 ^c^		66.7 ± 8.2 ^c^
10		86.7 ± 8.2 ^d^		83.3 ± 5.5 ^d^
1.5	2 (3.2 ± 1.3 g)	21.7 ± 7.5 ^a^	5 (8.0 ± 1.1 g)	23.3 ± 8.2 ^a^
2.5		46.7 ± 8.2 ^b^		43.3 ± 6.3 ^b^
5.0		63.3 ± 5.2 ^c^		63.3 ± 5.2 ^c^
10		81.7 ± 9.8 ^d^		86.7 ± 8.2 ^d^
1.5	3 (3.1 ± 0.5 g)	20.0 ± 6.3 ^a^	6 (8.2 ± 0.6 g)	18.3 ± 7.5 ^a^
2.5		43.3 ± 5.2 ^b^		46.7 ± 5.2 ^b^
5.0		65.0 ± 5.5 ^c^		65.0 ± 8.4 ^c^
10		85.0 ± 10.5 ^d^		85.5 ± 8.5 ^d^

Means with different letters within each challenge test indicate significant differences at P ≤ 0.05 by ANOVA and Tukey’s Honest Significant Difference test.

**Fig. 2:**
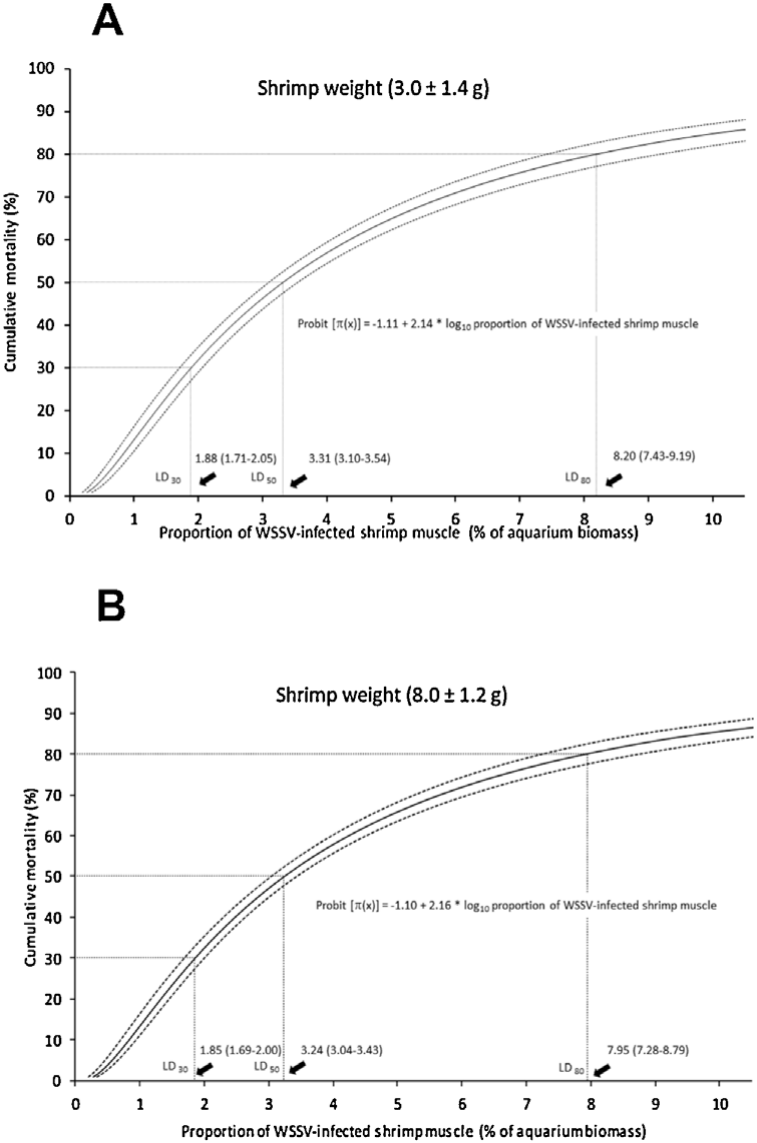
Probit analysis of cumulative mortality after 10 days post-infection as a function of the proportion (% of aquarium biomass) of WSSV-infected shrimp muscle (95% CI) supplied during the challenge tests for shrimp weights of 3.0 ± 1.4 g (A) and 8.0 ± 1.2 g (B). Estimated lethal doses 30, 50 and 80 (LD_30_, LD_50_ and LD_80_) for the proportion of WSSV-infected shrimp muscle to obtain cumulative shrimp mortalities of 30, 50 and 80% after 10 days post-infection are also showed. π(x) = Probability cumulative mortality.

Cumulative mortality significantly increased following a probit relationship with the proportion of infected-noninfected shrimp tissues (P < 0.001) across shrimp weight groups (Fig. 2). Estimated lethal doses were similar within and between shrimp weight ranges and their 95% confidence intervals (CI) overlapped (Fig. 2). Fig. 2 remarks such similarity and CI overlapping for LD_30_, LD_50_ and LD_80_, indicating absence of significant differences between shrimp weighing 3 and 8 g. Probability of cumulative shrimp mortality (Fig. 2) was estimated according to Eq. (1).

(1) Probability of cumulative mortality = P (z ≤ probit [π(x)])

Where, z = standard normal z-score at which the left-tail probability equals π(x).

**Fig. 3:**
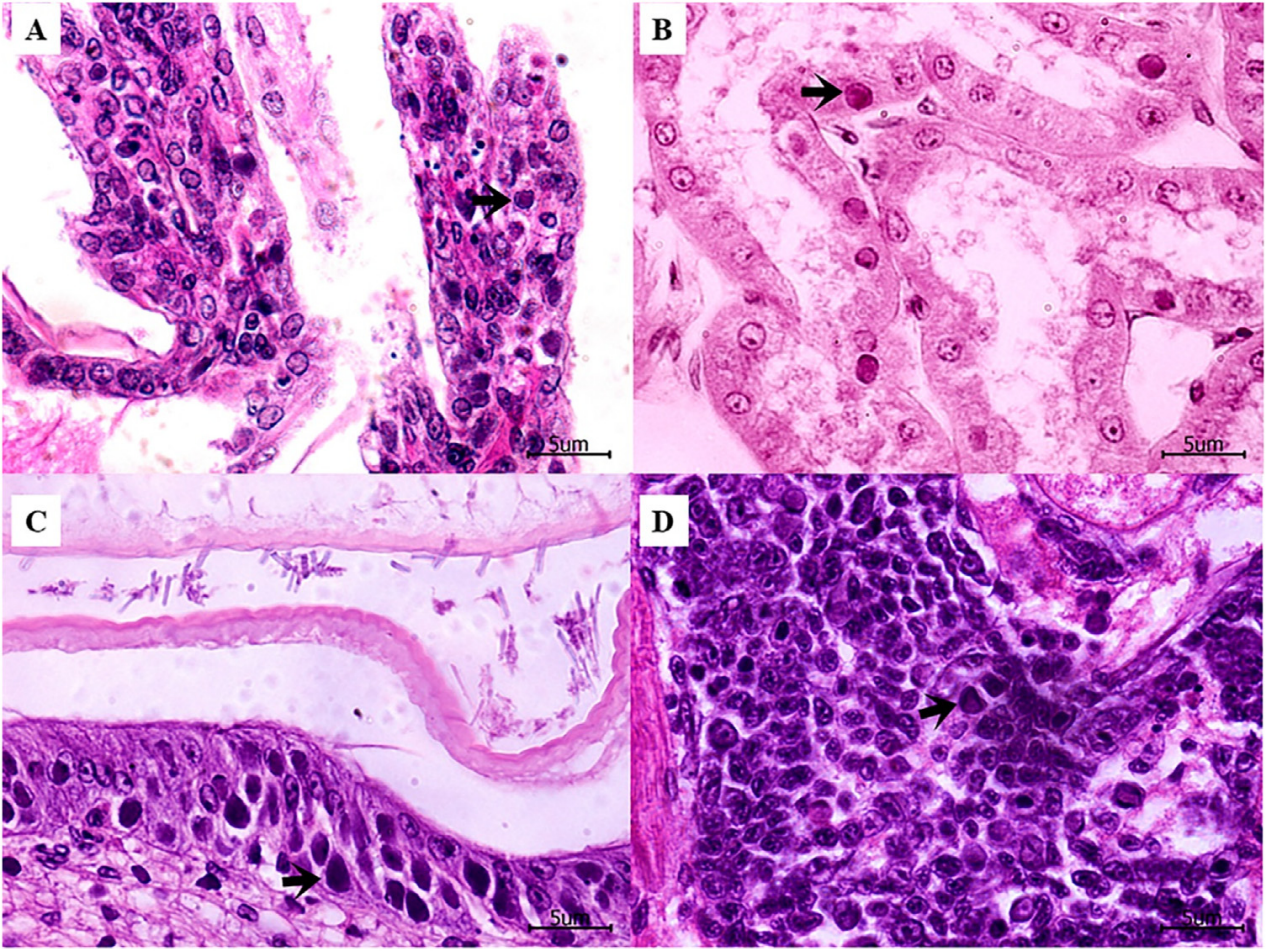
Hematoxylin and eosin-stained histological sections of moribund *P. vannamei* shrimp collected at 46 h post-exposure in the treatment where the viral papilla was prepared by the combination of 2.5% of WSSV-infected and 7.5% of noninfected tissues, which viral load was 1.13 × 10^7^ copies μg^−1^. Shrimp shows white spot disease (WSD) pathology caused by WSSV infection and characterized by cells with intranuclear inclusion bodies (arrow) in (A) gills, (B) antennal gland, (C) stomach epithelium and (D) hematopoietic tissue 40×. Scale bar = 5 μm.

In all challenge tests, the PCR and histopathology analyses (Fig. 3) confirmed that moribund shrimps in all infected treatments were severely affected with WSSV.

**Fig. 4:**
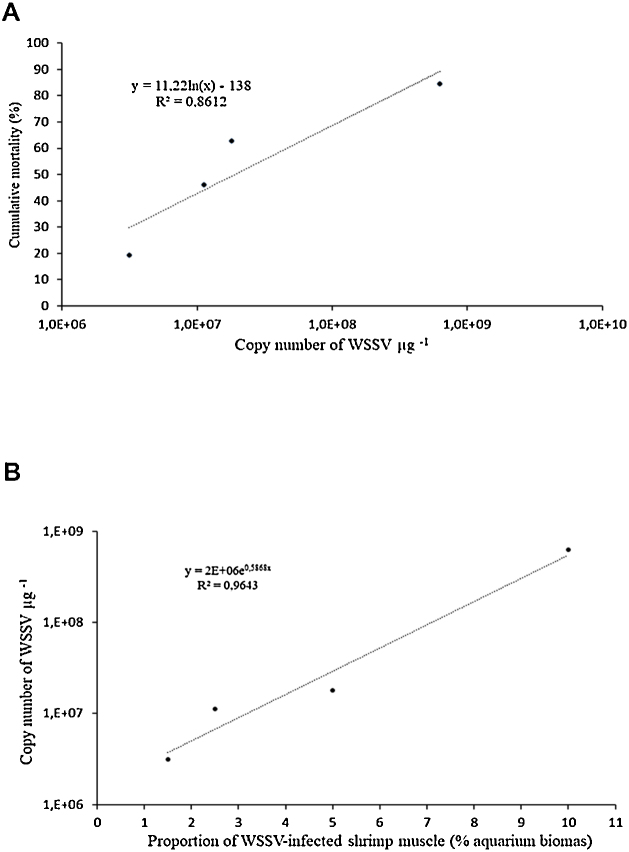
(A) Cumulative mortality of *P. vannamei* shrimp after 10 days post-infection in six challenge tests *versus* copy number of WSSV quantified by PCR-real time and (B) Copy number of WSSV *versus* the proportions of WSSV-infected muscle tissues, for the four viral papillae used in this study.

The combination of WSSV-infected and non-infected shrimp muscle provoked different mortality levels, which increased with the viral load (Fig. 4A). The 1.5, 2.5, 5.0 and 10.0% of WSSV-infected shrimp muscle (% of aquarium biomass) used in the viral papillae contained 3.14 × 10^6^, 1.13 × 10^7^, 1.80 × 10^7^ and 6.29 × 10^8^ viral copies per μg, respectively (Fig. 4B).

**Fig. 5:**
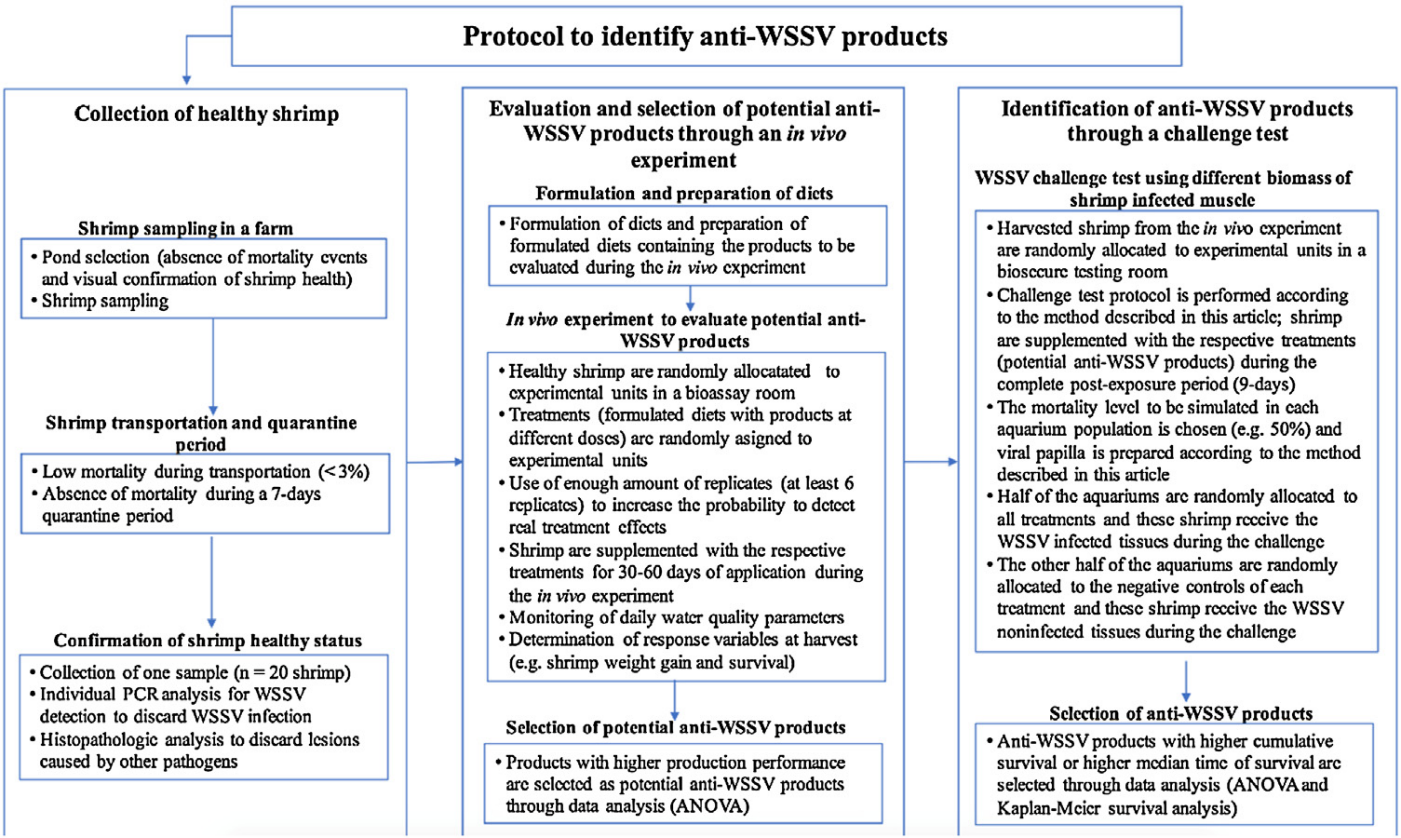
Scheme of a complete protocol to identify anti-WSSV products that includes the use of the challenge test method described in this article.

Once different combinations of WSSV-infected and noninfected shrimp tissues, and the correspondent mortalities levels, are identified, antiviral WSSV products can be identified using a complete protocol that includes the use of the method described in this article (Fig. 5). Briefly, the protocol consists of a process of three main steps. In the first step, healthy shrimp are collected from a pond without reports of mortality events. The health status of the collected shrimp after a quarantine period of 7 days is verified by PCR and histopathology analysis using a sample. In the second step, healthy shrimp are used for the evaluation and selection of potential anti-WSSV products over the production variables during an *in vivo* experiment. Previously, diets are formulated and prepared to contain the products to be evaluated during the *in vivo* experiment. Shrimp are fed with the formulated diets during a period of 30–60 days and production variables are determined at harvest. Potential anti-WSSV products with the best performance during the *in vivo* experiment are selected and used for the third step, where anti-WSSV products are identified through a challenge test. In this step, the mortality level to be simulated in each aquarium population is choose and viral papilla is prepared according to the method described in the article. Anti-WSSV products with higher cumulative survival and/or higher median time of survival during the challenge test are selected and can be used for further evaluation in ponds.

## Additional information

Anti-WSSV products are usually evaluated through intramuscular injection, immersion, oral inoculation and feeding of shrimp with infected tissues challenge tests [11,15,16]. Intramuscular injection challenge tests are effective to obtain high and reproducible mortality levels [12,17]. However, this form of infection does not occur in nature, as viral particles are not confronted with shrimp natural barriers [18]. Unlike, the *per os* challenge test simulates one of the main natural routes of WSSV infection [19]. Intubation is an oral challenge test, where shrimp are individually oral inoculated with known and similar virus amounts and therefore have the advantages of obtaining a low mortality variability and the results of different studies can be compared [15]. In this study we focused on the *per os* challenge test because it reproduces natural infections in a shrimp farm (cannibalism). First *per os* WSSV challenge test of *P. vannamei* shrimp using one dose of infected minced tissue was reported by Wang et al. [11]. Later, several other protocols of *per os* challenge tests have been performed, most of them performed with only one dose. Here we report a WSSV *per os* challenge test based on a dose-response model, which was standardized through the mortality responses to different amounts of WSSV-infected tissues. This method can simulate a wide and reproducible range of mortalities and is a simple and economic method to evaluate therapeutic antiviral products against WSSV by varying the proportion of WSSV-infected and noninfected tissues administered to susceptible shrimp.

The *per os* challenge test *via* feeding has been criticized because shrimp within treatments do not ingest equal amounts of infected tissue, thus leading to an increased variation of the response variable (mortality) and pathogens are less infectious than intramuscular inoculation procedures [12]. However, we demonstrated that a wide range of shrimp mortalities with low variability at each proportion of WSSV-infected tissue can be obtained (with an approximately standard deviation of 2.5% among challenge tests). During feasting, viral particles could be lixiviated into the water permitting other routes of infection. In this form, some organs, including antennal gland of shrimp could be infected, since according to immunohistochemistry analysis, this is one of the first shrimp organs to be infected [20]. In fact, a new WSSV infection method of shrimp has been developed by inoculating the pathogen *via* the antennal gland [21]. In either transmission case, we can simulate a wide and reproducible mortality range with the method described in this article.

On the other hand, water temperature affects WSSV infection, WSD progress and therefore shrimp mortality [22,23]. In our study, water temperature fluctuated over a wide range from 25 °C to 28 °C, and although securely this was other source of the error observed in the mortality response, similar levels of mortalities within and between challenge tests were observed, reflecting that we reproduced similar mortalities levels through time. However, it is recommendable that the calibration process will be performed avoiding great temperature variation to minimize the error sources. Another advantage of the method described here is its simplicity, as it does not require virus purification, tampon solutions, trained personnel to manipulate individual shrimp, PCR-real time thermocycler and other specialized equipment. A 20 °C freezer will be enough to maintain infected material viable.

Fig. 4A shows an R^2^ equal to 0.86, which indicates that 86% of the mortality variability is explained by the number of WSSV copies. With the information that we have is difficult to explain the sources of the rest of variability, which could include differences between experiments of water temperature, shrimp size, shrimp genetic, viral load, among others. It is important to consider, that we did not measure the viral load in each experiment, because we focus on the relation between mortality and the combination of infected and noninfected tissues, rather than in the quantification of the viral load. However, the treatment containing 10% of the WSSV infected tissue can be considered as a control, as in this treatment the papilla was not combined with noninfected tissues. The mortality of this treatment presented low variability in all six challenge tests (in average 85%, range = 83.3%–86.7%). Therefore, we deduce that all used batch presented similar viral loads and probably other sources that we did no measure could explain the rest of the variability.

Shrimp producers can easily evaluate antiviral products against WSSV on their own by simulating similar infecting conditions usually occurring in the pond using the methodology described in this article. Nevertheless, the proportions of WSSV-infected shrimp muscle can vary depending of the virulence of the WSSV strain. Hence, a similar calibration process reported in this study could be performed before the method implementation.
